# Infant Feeding Practices in Ethiopia: Birth Cohort Study in Five Regions

**DOI:** 10.1111/mcn.13804

**Published:** 2025-01-31

**Authors:** Amare Tariku, Kassahun Alemu, Joanna Schellenberg, Tanya Marchant, Della Berhanu, Seblewengel Lemma, Atkure Defar, Theodros Getachew, Zewditu Abdissa, Tadesse Guadu, Solomon Shiferaw, Girum Taye, Meseret Zelalem, Lars Åke Persson

**Affiliations:** ^1^ Institute of Public Health, College of Medicine and Health Sciences University of Gondar Gondar Ethiopia; ^2^ Department of Disease Control, Faculty of Infectious and Tropical Diseases London School of Hygiene and Tropical Medicine London UK; ^3^ Health System and Reproductive Health Research Directorate Ethiopian Public Health Institute Addis Ababa Ethiopia; ^4^ School of Public Health Addis Ababa University Addis Ababa Ethiopia; ^5^ Maternal, Child & Adolescent Health Service Lead Executive Office Federal Ministry of Health Addis Ababa Ethiopia

**Keywords:** breastfeeding, complementary feeding, performance monitoring for action study, sugary food consumption

## Abstract

Appropriate infant feeding is crucial to ensure optimal child growth and survival. We aimed to assess infants' breastfeeding and complementary feeding practices from 0 to 12 months in Ethiopia. This study was a secondary analysis of data from the Ethiopia Performance Monitoring for Action panel study performed from July 2020 to August 2021. One thousand eight hundred and fifty infants were included from five Ethiopian regions: Addis Ababa City Administration, Oromia, Amhara, Afar, and Southern Nations, Nationalities, and Peoples Regions. Appropriate infant feeding practices were assessed using the World Health Organization measurement criteria and descriptive analysis. One‐year‐old infants were considered to have a diversified diet if they had complementary feeding comprising five or more food groups. Two‐thirds (67%, 95% CI: 63, 71) of newborns were put to the breast within 1 h after delivery. The median duration of exclusive breastfeeding was 6.5 months, and 69% (95% CI: 67, 71) were exclusively breastfed at 5 months. Almost all (97%; 95% CI: 96, 98) were still breastfeeding at 12 months. Sixteen percent (95% CI: 13, 19) of infants (boys 15%, girls 16%) aged 12 months had a diversified diet, and 49% (95% CI: 44, 55) consumed sugary foods or beverages. Most Ethiopian infants had appropriate breastfeeding practices, while almost all had poor‐quality complementary food at 1 year. Increasing access to high‐quality education on infant feeding is crucial to maintaining and enhancing appropriate breastfeeding practices and complementary food quality. Intensifying poverty reduction efforts are essential to improve infants' dietary diversity and nutrient‐dense food consumption.

## Summary

- Two‐thirds of Ethiopian infants had initiated breastfeeding within 1 h after birth.
- The coverage of exclusive breastfeeding at 5 months of age and continued breastfeeding at 12 months of age were in line with the global target for exclusive breastfeeding (≥ 70%) and WHO recommendations.
- Most infants aged 12 months did not have a diversified diet, and half consumed sugary foods or beverages.
- Intensifying infant feeding counselling is crucial to increase appropriate breastfeeding practices and to improve the quality of complementary feeding.
- Strengthening the national food insecurity reduction interventions is critical to enhancing the diversity and quality of infants' complementary food.

## 1 Introduction

The Sustainable Development Goals (SDGs) aim to end child hunger and reduce under‐five child deaths to less than 25 per 1000 live births (UN 2015). Appropriate breastfeeding and complementary feeding are cost‐effective means to ensure optimal child growth and survival (Bhutta et al. 2013). The WHO recommends initiating breastfeeding within 1 h after birth, exclusive breastfeeding up to 6 months of age, and continued breastfeeding for an additional 18 months or beyond. After 6 months of exclusive breastfeeding, infants should be introduced to safe and adequate complementary foods (WHO 2021). Globally, there is a target to increase the coverage of exclusive breastfeeding at 5 months to 70% by 2030 (Shekar et al. 2017).

Inappropriate breastfeeding and complementary feeding continue to be critical global nutrition challenges. Worldwide, less than one‐third of infants aged 4–6 months were exclusively breastfed, and half (52%) initiated breastfeeding within 1 h after birth (Wu et al. 2021). Only 17% of children aged 6–23 months in 80 low‐ and middle‐income countries had complementary feeding fulfilling the minimum diet diversity and meal frequency (Gatica‐Domínguez et al. 2021). In sub‐Saharan African countries, only a quarter was exclusively breastfed at 4–6 months of age (Wu et al. 2021), and the proportion of children 6–23 months getting a minimum acceptable diet was 13%, which was lower than the global average (Gatica‐Domínguez et al. 2021). According to the 2019 mini‐Demographic and Health Survey, almost all (96%) of Ethiopian infants were breastfed at some point, and 59% of those under the age of 6 months were exclusively breastfed in the 2 years before the survey (EPHI and ICF 2019). Infants and young children (6–23 months) were also found to have poor‐quality complementary food (Alemu et al. 2022). Poor access to and quality of infant nutrition education and counselling services have been mentioned as barriers to appropriate breastfeeding and complementary feeding practices (Abebe et al. 2016). Mothers' misconceptions about colostrum and the provision of prelacteal feeds may contribute to delayed initiation of breastfeeding. Perceived insufficiency of breast milk may lead to an early cessation of exclusive breastfeeding (Mekonnen et al. 2018; Gizaw et al. 2023). Lack of nutrition literacy on the benefits of dietary diversity and fruit and vegetable consumption, expensiveness of animal products, and food shortage have been listed as barriers to appropriate complementary feeding (Gizaw et al. 2023). Aggressive marketing, economic access, and less time required to prepare food were associated with children's consumption of sugary foods and beverages (Tadesse et al. 2024).

Following the WHO recommendations (WHO 2021), Ethiopia implemented infant and young child feeding intervention packages to improve children's nutrition and survival. The National Food and Nutrition Strategy envisioned an increased coverage of appropriate breastfeeding and complementary feeding practices as essential to breaking the intergenerational cycles of malnutrition. Assessing the coverage of appropriate infant feeding practices could help to evaluate Ethiopia's progress toward the global exclusive breastfeeding target at 5 months of age, that is, > 70%. These efforts could contribute to ending child hunger and reducing under‐five child mortality to less than 25 per 1000 live births by 2030 (UN 2015; Shekar et al. 2017). Available Ethiopian infant feeding studies measured breastfeeding practice at a single time point or using long recall periods (up to 3 years), which may reduce the accuracy of estimates (Potts et al. 2021; Gebremedhin et al. 2021). The previous nationally representative studies of complementary feeding have reported dietary diversity or specific nutrient groups for a wide age range, that is, 6–23 months (Demsash et al. 2022; Alemu et al. 2022). Consumption of sugary foods and beverages in early childhood was associated with a higher risk of overweight or obesity and related non‐communicable diseases (Ferretti and Mariani 2019). Despite this, few studies from local geographic areas have addressed infants' sugary food consumption (Tizazu et al. 2022; Jemere et al. 2023). Therefore, this birth cohort study aimed to comprehensively assess the initiation of breastfeeding within 1 h of birth and the duration of exclusive and continued breastfeeding in a nationally representative sample. We also aimed to measure infants' diet diversity, micronutrient intake, and sugar‐containing beverages and snacks consumption at 12 months of age.

## 2 Methods

### 2.1 Study Setting

Ethiopia is a low‐income country with an estimated population size of 120 million. The Ethiopian food consumption pattern is mainly based on starchy staples that contribute about two‐thirds of the daily food consumption (EPHI 2013). The Ethiopian health care system is a three‐tiered system, that is, the primary, secondary, and tertiary levels of health care. This study used data from the Performance Monitoring for Action (PMA) panel study, a collaborative project between Addis Ababa University and Johns Hopkins Bloomberg School of Public Health. The PMA study was conducted from July 2020 to August 2021 in six Ethiopian regions to evaluate the coverage and comprehensiveness of the continuum of reproductive, maternal, and newborn health and health services utilization in the first year after birth. Four of these study regions are the most populous and predominantly agrarian, that is, Oromia, Amhara, Tigray, and Southern Nations Nationalities and Peoples (SNNP) Regions. The others are the urban Addis Ababa City Administration and the pastoralist Afar region (Qian, Wood et al. 2021).

### 2.2 Study Design, Population, and Sampling

The PMA study combined a cross‐sectional sample of women with a birth cohort panel study up to infants aged 12 months. All women 15–49 years who were pregnant or within 6 weeks postpartum and permanent residents of households in study clusters, including those living with parents for delivery, were eligible for the panel study. Our analysis was based on infants of the study mothers. The PMA study used a two‐stage stratified cluster sampling. The study regions were purposefully selected. One‐hundred eighty‐four clusters or enumeration areas (EA) were randomly selected proportional to the population of the study regions. EAs were selected from urban and rural strata of Tigray, Amhara, Oromia, and SNNP regions. In the Afar region, only rural EAs were included, while only urban EAs were selected in Addis Ababa. As of November 2020, the fieldwork in Tigray was stopped due to armed conflict. Hence, due to the incompleteness of data, Tigray was excluded from our analysis (Qian, Wood et al. 2021). The five study regions represented about 85% of the Ethiopian population. Further details of the sampling procedure and study approach are published elsewhere (Zimmerman et al. 2020).

### 2.3 Data Collection and Quality Control

The panel data were collected using standard questionnaires developed using the 2016 PMA‐Maternal and Newborn Health survey tool (Zimmerman et al. 2020). Efforts were made to align the questionnaire with the priority indicators of the Federal Ministry of Health. Eligible women were interviewed at baseline to estimate the expected delivery date (using approximate gestational age) and to capture important demographic, socio‐economic, and reproductive health characteristics. Considering the expected delivery date, the data collectors (who resided in the community where data were being collected) checked whether women gave birth. After delivery confirmation, follow‐up interviews were planned, the first at about 6 weeks, the second at 6 months, and the third at twelve months after delivery. For 2 weeks, data collectors, supervisors, and regional coordinators were trained on interview techniques, tools, and survey protocols. This training was supported by 3 days of field testing of the electronic data collection using the Open Data Kit. Supervisors provided on‐site support to data collectors and did regular checks to enhance data quality. Based on the registered geographic coordinates of households, the central data management team checked that interviews were conducted within the study area. When potential errors were identified, the team communicated with the data collectors to make corrections.

### 2.4 Assessment of Infants' Breastfeeding and Complementary Feeding

The breastfeeding and complementary feeding practices assessment was based on standard WHO indicators (WHO 2021). At the first follow‐up interview, mothers were asked when they first put the newborn to the breast. Early breastfeeding initiation was defined as the proportion of newborns first put to the breast within 1 h after birth. Mothers were interviewed about the age at introduction of any other food in addition to breast milk at the second and third follow‐up interviews. Exclusive breastfeeding was defined as the percentage of infants fed exclusively on breast milk and no other food or fluid. The intake of medicines, oral rehydration solution, vitamins, and minerals was not included in the definition of exclusive breastfeeding. Otherwise, an infant 0–5 months was considered to have mixed feeding when fed breast milk plus any other additional food or liquid.

Continued breastfeeding was assessed at the first, second, and third panel interviews and reported as the proportion of infants who were fed breast milk within 24 h before the follow‐up interviews. Complementary feeding was assessed at the third follow‐up interview, including data on seven infant food groups (WHO 2021). The consumption of energy‐dense and low‐micronutrient‐containing foods was assessed. Feeding practices were shown as the proportion of infants who drank sweet beverages or ate sugary or savoury foods in the 24 h before the survey. Sweet beverages included all homemade or commercially produced and packed drinks flavoured with sweeteners or to which sugar was added. Sugary or savoury foods comprised homemade or commercially produced and packed sugary and fried foods, including fried samosa, donuts, chips, cake, pastries, sweet biscuits, or candies.

### 2.5 Analyses

Participation was displayed using a study flow diagram. Descriptive statistics with a 95% confidence interval (CI) were used to display infants' breastfeeding and complementary feeding practices. The frequencies and proportions were adjusted for sampling weights to ensure the sample represented all pregnant and postpartum women in the five Ethiopian regions. The sampling weight was the inverse probability of the enumeration area (primary sampling unit) and household (secondary sampling unit) selections. Household wealth scores were generated by principal component analysis based on ownership of livestock, assets, and house materials. The scores were categorized into quintiles from the poorest to the wealthiest. Infants' age was calculated by subtracting the date of the interview from the date of birth, and the median age of infants was also estimated for each follow‐up interview. The chi‐square test was used to analyse the socio‐demographic characteristics of participating and non‐participating infants. A sensitivity analysis was carried out to check whether the loss of follow‐ups affected the infant feeding indicators. The duration of exclusive breastfeeding was calculated based on the age when any other food was introduced. Life table analyses were used to generate exclusive breastfeeding curves and estimate the median exclusive breastfeeding duration in the total sample and different strata. Minimum dietary diversity was estimated as the percentage of infants aged 12 months who had complementary food made from five or more standard food groups the day before the survey. The vitamin A‐rich food intake was shown as the percentage of infants aged 12 months who consumed vitamin A‐rich plant (green leafy vegetables, yellow‐orange fruits, and vitamin A‐rich vegetables or tubers) or animal‐based (organ meat, egg, and dairy) foods in the 24 h before the survey. The iron‐rich food intake was analysed based on the percentage of infants who ate organ meat, meat, fish, or seafood 24 h before the survey. Zero vegetable or fruit consumption was estimated as the percentage of infants aged 12 months who did not eat vegetables or fruits in the 24 h before the survey. All analyses were adjusted for clustering. Regional variations in infants' early initiation of breastfeeding, sugary food or beverage consumption, and diet diversity scores were examined using mixed‐effect binary logistic and Poisson regression models. Variation in the duration of exclusive breastfeeding by infants' sex was analysed using a log‐rank test.

### 2.6 Ethical Statement

This study was based on a secondary analysis of data from the PMA‐Ethiopia study, which was reviewed and approved by the Institutional Review Boards of Addis Ababa University, College of Medicine and Health Sciences, and the Johns Hopkins University Bloomberg School of Public Health.

## 3 Results

### 3.1 Participation

**Figure 1:**
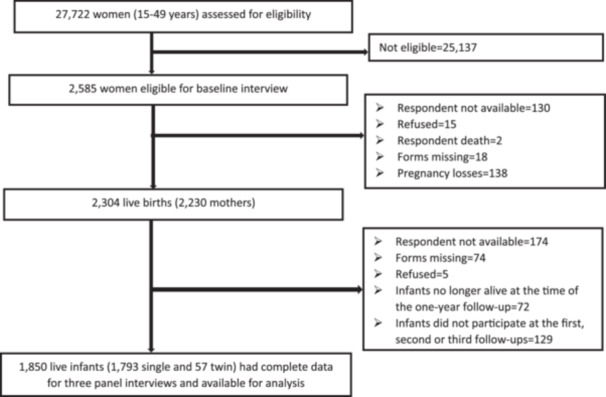
Study flow diagram illustrating mothers and their infants who completed the 1‐year postpartum follow‐up (July 2020–August 2021) in five Ethiopian regions, PMA Ethiopia panel study.

Twenty‐seven thousand seven hundred twenty‐two women of reproductive age (15–49 years) were assessed for eligibility, and 2585 fulfilled the eligibility criteria (pregnant or within 6 weeks after birth) in the baseline interview (Figure 1). The first and second follow‐up interviews were conducted at a median age of 1.7 months (inter‐quartile range [IQR]: 1.6 months) and 6.7 months (IQR: 2.0 months) after delivery, respectively. The third follow‐up was conducted at a median age of 12.2 months (IQR: 0.5 months). Two thousand three hundred four live births from 2230 mothers completed the first follow‐up interview. At 12 months, 1850 infants remained in the study. Thus, 454 infants were lost to follow‐up, mainly due to the unavailability of interviews, incomplete data, and infant death. There were statistically significant differences in socio‐demographic characteristics between participating and non‐participating infants (Table S1). The sensitivity analysis result showed that losses to follow‐up increased the prevalence of vitamin A‐rich food, animal protein‐rich food, and sugary food consumption while underestimating zero fruit or vegetable consumption (Table S2).

### 3.2 Socio‐Demographic Characteristics of Mothers and Infants

About half of the households had five or more family members (Table 1). One‐tenth of the mothers were aged 15–19 years, and almost all (98%) were married. Two‐fifths had no schooling and one‐third had a parity history of five children or more. Three‐fourth (77%) of the mothers had an antenatal care visit, 33% had four or more visits, and a small proportion (3%) had eight or more visits. About half gave birth at health facilities (55%), and one‐quarter received postnatal care within 2 days after delivery. One‐tenth of mothers had been counselled about exclusive breastfeeding, and 20% about infant dietary diversity. Very few (4%) reported counselling about not feeding sugary foods or beverages.

**Table 1:** Socio‐demographic characteristics of mothers and infants who completed a one‐year postpartum follow‐up, PMA Ethiopia panel study, July 2020 to August 2021 (*N* = 1850).

Characteristics	Unweighted *n* (%)	Weighted^a^ % (95% CI)
Region		
Afar	178 (10)	2 (1, 3)
Amhara	413 (22)	24 (21, 27)
Oromia	539 (29)	46 (41, 51)
Southern Nations, Nationalities and Peoples	506 (27)	24 (20, 28)
Addis Ababa	214 (12)	4 (3, 5)
Residence		
Urban	640 (35)	21 (16, 27)
Rural	1210 (65)	79 (73, 84)
Household size (*n* = 1849)		
One to four	961 (52)	49 (45, 53)
Five to six	561 (30)	31 (29, 34)
Seven to sixteen^b^	327 (18)	20 (17, 24)
Household wealth quintiles (*n* = 1849)		
1 (Poorest)	353 (19)	21 (16, 27)
2	297 (16)	20 (17, 23)
3	312 (17)	21(18, 24)
4	331 (18)	19 (16, 24)
5 (Wealthiest)	556 (30)	19 (14, 24)
Mothers age (in years) (*n* = 1849)		
15–19	145 (8)	9 (7, 10)
20–24	433 (23)	23 (21, 26)
25–29	624 (34)	33 (30, 35)
30–34	364 (20)	20 (17, 22)
35–47	283 (15)	17 (15, 19)
Marital status (*n* = 1849)		
Currently married^c^	1817 (98)	98 (97, 99)
Currently unmarried^d^	32 (2)	2 (1, 3)
Religion (*n* = 1849)		
Orthodox	708 (38)	36 (31, 43)
Muslim	614 (33)	34 (26, 43)
Protestant and others^e^	527 (29)	29 (23, 36)
Education (*n* = 1849)		
No schooling	753 (41)	43 (38, 48)
Primary school	671 (36)	40 (36, 44)
Secondary school or above	425 (23)	17 (14, 21)
Parity^f^ (*n* = 1846)		
1	321 (17)	16 (14, 18)
2	427 (23)	21 (19, 24)
3–4	550 (30)	29 (27, 31)
≥ 5	548 (30)	34 (30, 38)
Number of antenatal care visits (*n* = 1848)		
0	487 (26)	23 (19, 29)
1	122 (7)	9 (6, 12)
2	199 (11)	13 (11, 16)
3	348 (19)	22 (19, 25)
≥ 4	691 (37)	33 (28, 38)
Place of delivery (*n* = 1849)		
Health facility	1097 (59)	55 (49, 61)
Home	752 (41)	45 (39, 51)
Received postnatal care within two days		
No postnatal care	1050 (57)	61 (56, 66)
Received ≤ 2 days	506 (27)	27 (23, 31)
3–7 days	162 (9)	6 (4, 7)
8–28 days	132 (7)	6 (5, 8)
Received counselling about exclusive breastfeeding at the first follow‐up interview	301 (16)	14 (11, 17)
Received counselling on complementary feeding after 6 months of age		
Dietary diversity	390 (21)	20 (16, 23)
Giving animal source food	257 (14)	13 (11, 16)
Meal frequency	176 (10)	9 (7, 11)
Not feeding sugar‐sweetened beverages	91 (5)	4 (3, 5)
Child sex		
Boy	949 (51)	51 (49, 54)
Girl	901 (49)	49 (46, 51)

^a^ Analysis adjusted for enumeration area and household sampling weights.

^b^ Eighteen households had 11–16 members.

^c^ Forty‐three living with a partner.

^d^ Divorced, never married and widowed.

^e^ Others (Catholic, Wakefeta, Traditional).

^f^ Fourteen mothers had 10–14 parity history.

### 3.3 Breastfeeding Practice of Infants Aged 0–12 Months

**Figure 2:**
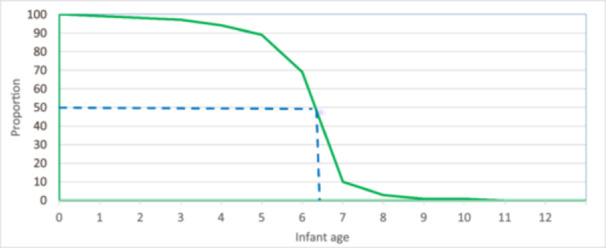
Proportion of infants exclusively breastfed by age. The median duration of exclusive breastfeeding marked. PMA Ethiopia panel study, July 2020 to August 2021 (*N* = 1849).

All mothers initiated breastfeeding during the first week. Two‐thirds (67%) of infants were put to the breast within 1 h after birth (no difference between girls and boys), 26% initiated breastfeeding after 1 h but within a day (no difference between girls and boys), and 7% initiated breastfeeding after the first day. Almost all infants were still breastfed at the second (98%) and third (97%) follow‐up interviews. The median duration of exclusive breastfeeding was 6.5 months, without any difference between boys and girls. Table 2 and Figure 2 show the duration of exclusive breastfeeding and age at the introduction of complementary feeding. Most (94%) of the infants were exclusively breastfed at 3 months, and 69% were exclusively breastfed at 5 months. Only 3% were still exclusively breastfed at 7 months. There was no significant difference in the duration of exclusive breastfeeding by infants' sex.

**Table 2:** Duration of exclusive breastfeeding among infants who completed 1‐year follow‐up, PMA Ethiopia panel study, July 2020 to August 2021 (*N* = 1849).

Age interval in months	Number of exclusively breastfed at start of interval	Number started other food	Proportion exclusively breastfed at start of interval	95% CI
0–0.9	1849	27	99	98, 99
1.0–1.9	1822	19	98	97, 98
2.0–2.9	1803	17	97	96, 98
3.0–3.9	1786	48	94	93, 95
4.0–4.9	1738	101	89	87, 90
5.0–5.9	1637	368	69	67, 71
6.0–6.9	1269	1090	10	8–11
7.0–7.9	179	117	3	2–4
8.0–8.9	62	40	1	0, 2
9.0–9.8	22	10	1	0, 1
10.0–10.9	12	6	0	0
11.0–11.9	6	3	0	0
12.0–12.9	3	3	0	0

### 3.4 Infants' Complementary Feeding at 12 Months

Only 16% (95% CI: 13, 19) of infants received complementary food at 12 months that fulfilled the minimum dietary diversity, that is., receiving items from five food groups or more (Table 3). Dairy consumption was reported in less than half of the infants. Only 6% ate meat, fish, or poultry, and one‐fifth had eggs 24 h before the interview. Less than two‐thirds received complementary foods containing animal protein. Very few (6%) ate an iron‐rich diet, that is, meat, organ meat or fish. Below two‐thirds of infants ate complementary foods from plants or animals rich in vitamin A, including dark green leafy vegetables, yellow and orange fruits, dairy, eggs, and organ meat. Two‐thirds of infants did not get any fruits or vegetables 24 h before the survey. Half of the infants had sugary foods or beverages consumption.

**Table 3:** Complementary feeding practices of infants aged 12 months, PMA Ethiopia panel study, July 2020 to August 2021 (*N* = 1850).

Complementary feeding practices using 24‐h recall	Weighted^a^ % (95% CI)
Dietary diversity	
Minimum diet diversity^b^	16 (13, 19)
Breast milk	97 (96, 98)
Grains, roots, and tubers	94 (91, 95)
Pulses and nuts	43 (37, 48)
Dairy products	45 (40, 50)
Meat, fish, poultry, or organ meats	6 (5, 7)
Eggs	20 (17, 24)
Vitamin A‐rich fruits and vegetables	25 (22, 29)
Other fruits and vegetables	8 (6, 9)
Animal protein intake^c^	55 (51, 60)
Micronutrient‐rich food consumption	
Iron‐rich diet	6 (4, 8)
Vitamin A‐rich food intake	62 (58, 66)
Unhealthy food consumption	
Sugary food or beverage consumption	
Zero fruit or vegetable	65 (61, 69)
Sweet beverage	39 (34, 44)
Sugary and savoury food	21 (17, 25)
Sugary food or beverage consumption	49 (44, 55)

^a^ Analyses adjusted for enumeration area and household sampling weights.

^b^ Five or more food groups.

^c^ Flesh, dairy and egg.

### 3.5 Regional Variations in Infants' Breastfeeding and Complementary Feeding

Table 4 shows the regional variation in infants' breastfeeding and complementary feeding practices. Afar region had a significantly lower frequency of early initiation of breastfeeding compared to other Ethiopian regions. Two‐fifths of the infants were put to the breast within 1 h after birth in Afar, while about three‐quarters initiated breastfeeding early in Oromia. Early initiation of breastfeeding was reported in two‐thirds of infants in Addis Ababa, Amhara, and SNNP Regions. There was no regional variation in exclusive breastfeeding duration. Two‐fifths of infants in Addis Ababa had complementary feeding at 1 year, meeting the minimum diet diversity, while only 2% in Afar had a diversified diet. One‐tenth of infants in the Amhara region and below one‐fifth in Oromia and SNNP had a diversified diet. Infants' sugary foods or beverages consumption was significantly higher (74%) in Addis Ababa City administration as compared to other regions.

**Table 4:** Regional variations in breastfeeding and complementary feeding practices of infants aged 12 months, PMA Ethiopia panel study, July 2020 to August 2021 (*N* = 1849).

Feeding indicators	Afar^a^	Amhara^a^	Oromia^a^	SNNP^a^	Addis Ababa^a^	*p*‐value
Early initiation of breastfeeding^b^ (%)	44 (26, 63)	59 (50, 68)	72 (66, 78)	67 (56, 76)	66 (56, 74)	< 0.001
Median duration of exclusive breastfeeding in months^c^	6.5	6.5	6.5	6.5	6.5	0.815
Diet diversity score at 12 months of age^d^						< 0.001
0	1 (0.1, 5.6)	0	0	0	0	
1	6 (3, 10)	4 (2, 6)	2 (1, 4)	5 (3, 7)	2 (0.4, 4.3)	
2	42 (35, 50)	15 (12, 19)	19 (16, 22)	21 (17, 25)	8 (5, 12)	
3	43 (36, 50)	46 (40, 49)	35 (31, 39)	32 (28, 36)	20 (15, 26)	
4	7 (4, 12)	24 (20, 29)	29 (25, 33)	22 (19, 26)	30 (25, 37)	
5	2 (1, 6)	9 (7, 12)	11 (9, 14)	12 (9, 15)	23 (18, 30)	
6	0	3 (2, 6)	4 (3, 6)	7 (5, 10)	14 (10, 20)	
7	0	0.2 (0.02, 2)	1 (0.2, 2)	0.2 (0.02, 1.4)	3 (2, 7)	
8	0	0	0	0	0	
Sugary food or beverage consumption at 12 months of age^b^ (%)	51 (43, 58)	53 (48, 58)	53 (49, 57)	48 (43, 52)	74 (68, 80)	< 0.001

^a^ Weighted % (95% CI).

^b^ Mixed‐effect binary logistic regression.

^c^ Logrank test.

^d^ Mixed‐effect Poisson regression.

## 4 Discussion

### 4.1 Summary of Findings

Two‐thirds of infants were initiated on breastfeeding within 1 h after birth, and all infants were breastfed within the first week. Seven out of ten infants were exclusively breastfed at 5 months of age, and almost all were still breastfeeding at 12 months of age. According to 24‐h recalls, only a small number of infants had a diversified diet at 1 year, two‐thirds had vitamin A‐rich food, but very few had an iron‐rich food intake. One‐third consumed fruits or vegetables, and half of the infants consumed sugary foods or beverages. Thus, most infants were breastfed in line with global and national recommendations, while few infants had an appropriate diversity of complementary foods at 12 months.

### 4.2 Strength and Limitations

This study was based on the analysis of data from five regions representing 85% of the Ethiopian population, including urban, agrarian, and pastoralist populations (Zimmerman et al. 2020). Previous studies measured either breastfeeding or complementary feeding practices and were based on longer recall periods, that is, up to 3 years (Potts et al. 2021; Gebremedhin et al. 2021). Our prospective evaluation of infant breastfeeding practices could improve the accuracy of estimates by minimizing recall bias. Some participants may have misunderstood early initiation of breastfeeding as the time when the newborn sucks breast milk rather than when a mother puts her baby to the breast to initiate breastfeeding (Salasibew et al. 2014). Such misunderstanding could lead to an underestimation of the coverage of early breastfeeding initiation. This study used the WHO‐recommended multiple breastfeeding and complementary feeding indicators, which provide a comprehensive picture of infant feeding practices from birth to first birthday (WHO 2021). Infants' complementary feeding was assessed using 24‐h recall, which could enhance mothers' memory in reporting the food items consumed by infants. However, a single 24‐h recall does not necessarily show the infants' usual dietary behaviour (Kennedy et al. 2011). This could potentially lead to an underestimation of dietary diversity. However, on the group level, 24‐h recalls reflect the usual pattern quite well. In addition to applying the WHO infant feeding measurement approaches, efforts were made to enhance the data quality through training and supervision of the data collectors, who resided in the community where data were collected. Using such data collectors increased their familiarity with mothers and the study setting context.

### 4.3 Breastfeeding Practices

Although WHO recommends all newborns initiate breastfeeding within 1 h after birth, one‐third of the newborns in our study were not put to the breast within 1 h after birth. However, all newborns were breastfed within a few days. The coverage of exclusive breastfeeding (69%) at 5 months of age was in line with the 2030 global target (≥ 70%) (Shekar et al. 2017). The observed coverage of continued breastfeeding at 12 months of age (98%) was also in agreement with WHO recommendations (WHO 2021). Our result of early initiation of breastfeeding was higher than the global (52%) as well as the African (56%) averages. Similarly, the coverage of exclusive breastfeeding at 5 months of age was higher than the global (32%) and African region (27%) averages at 4–5 months of age (Wu et al. 2021). Similar levels of early initiation (72%), exclusive breastfeeding at under 6 months of age (59%) (EPHI and ICF 2017), and continued breastfeeding at 12 months of age (92%) were reported from the Ethiopian Demographic and Health Surveys (EPHI and ICF 2019). Reportedly, Ethiopian mothers may discard the first breast milk or colostrum, assuming that it is dirty or not good for infants' health (Mekonnen et al. 2018; Gizaw et al. 2023). This perception may lead to providing prelacteal feeds immediately after birth, such as raw butter, with the intention of smoothening the newborn's gut. These habits might explain why one‐third of Ethiopian newborns had delayed initiation of breastfeeding. The likelihood of early cessation of exclusive breastfeeding was higher among Ethiopian mothers who provided prelacteal feeds to their newborns or perceived their milk supply as insufficient to meet their infant's nutritional needs (Mekonnen et al. 2018; Gizaw et al. 2023). Strengthening the implementation of evidence‐based interventions, such as the Baby Friendly Health Facility Initiative, which is prioritized in the Ethiopian National Food and Nutrition Strategy, may enhance the early initiation and exclusive breastfeeding of young infants (Von Seehausen et al. 2023). Community‐based breastfeeding education and support intervention help to reach women who are socially and geographically disadvantaged or have lesser access to facility‐based interventions (Dyson et al. 2006). Evidence showed that the provision of breastfeeding education and support by community health workers and non‐health professionals enhanced the coverage of early initiation and exclusive breastfeeding in low‐ and middle‐income countries (Dyson et al. 2006; Mituki‐Mungiria et al. 2020). Mothers' experience‐sharing or peer‐counselling may also enhance exclusive breastfeeding (Buckland et al. 2020). The Ethiopian volunteer community health workers (Women's Development Groups) prenatal and postnatal home visits for breastfeeding education and support increased rural mothers' early initiation and exclusive breastfeeding practices (Abdulahi et al. 2021). Targeting the mother‐father dyads and supporting breastfeeding education with mobile or digital health interventions may also enhance appropriate breastfeeding practices (Qian, Wu et al. 2021; Gebremariam et al. 2023).

### 4.4 Complementary Feeding Practices

Most Ethiopian infants had poor complementary feeding practices at 12 months. Only a small group of infants had complementary food meeting the minimum dietary diversity criterion. Findings showed low consumption of iron‐rich foods. The observed poor diet diversity in this study was lower than those earlier reported from Southern Asia (21%) and Africa (19%–36%) (Gatica‐Domínguez et al. 2021). The 2019 Ethiopia Demographic and Health Survey had a similar level of diet diversity (14%) among children aged 6–23 months (EPHI and ICF 2019). The low dietary diversity and poor consumption of iron‐ and vitamin A‐rich foods could make infants prone to undernutrition, including micronutrient deficiencies (Bhutta et al. 2013; UN 2015; Keats et al. 2021). The high burden of household food insecurity in Ethiopia could underlie the observed poor complementary feeding practices (International Rescue Committe 2023). Countries' Gross Domestic Product is reportedly associated with infants' diet diversity. Infants of the better‐off families have higher dietary diversity, animal food, and fruit and vegetable consumption (Gatica‐Domínguez et al. 2021). A lack of nutrition literacy about the needs and benefits of providing a variety of food to infants to support growth and development could also underlie the observed poor dietary diversity (Tadesse et al. 2024). Facility‐ and community‐based infant nutrition education and support interventions could positively influence infants' appropriate breastfeeding and diet diversity (Ahmed et al. 2021; Gizaw et al. 2022). Addressing the reported lack of access to infant feeding counselling and education could help to maintain and further enhance appropriate breastfeeding practices and improve dietary diversity and micronutrient‐rich food intake. Community health workers' cooking demonstration and complementary feeding education in Ethiopia or elsewhere may enhance mothers' infant nutrition literacy, infants' dietary diversity, and meal frequency (Ahmed et al. 2021; Waswa et al. 2015; Rahman et al. 2023). Delivery of complementary feeding awareness creation and behavioural change interventions via mobile health technologies have enhanced infants' animal food consumption and the quality of complementary feeding (Downs et al. 2019).

Half of this study's infants had consumed sugary foods or beverages in the last 24 h. Infants' and young children's consumption of sugary foods or beverages is becoming prevalent in Asian and African countries (Huffman et al. 2014). Increased access, aggressive marketing, and low health literacy regarding these unhealthy foods may explain the early start of consumption of such food items (Cartwright et al. 2023). Dietary counselling for mothers may reduce infants' sugary food or beverage consumption (Vitolo et al. 2012). Sugary food or beverage consumption undermines nutrient density or quality of complementary food, that is, leading to inadequate intake of minerals and vitamins (Mumena 2021). It is also associated with a higher risk of becoming overweight or obese (Ferretti and Mariani 2019).

### 4.5 Regional Variations in Breastfeeding and Complementary Feeding Practices

Findings showed significantly higher early initiation of breastfeeding in Addis Ababa and other regions compared to Afar Region. The considerable difference in early initiation of breastfeeding between Afar and other regions could be explained by tradition of prelacteal feeds (Tsegaye et al. 2019; Potts et al. 2021). Infants' diet diversity was significantly higher in Addis Ababa compared to predominantly agrarian and pastoralist regions. Better employment and income diversification, as well as essential attributes of household food security in urban settings of Ethiopia, could explain the reported higher diet diversity in Addis Ababa (Adem et al. 2018; Etea et al. 2019). However, infants in Addis Ababa also had higher consumption of unhealthy food, that is, sugary food or beverages. Similar consumption of unhealthy food or beverages was reported in other urban settings of Ethiopia (Jemere et al. 2023). High access to and promotion of unhealthy foods in Addis Ababa could underlie the observed consumption of sugary foods and beverages.

## 5 Conclusions

Our findings showed high coverages of early initiation of breastfeeding, exclusive breastfeeding at 5 months of age, and continued breastfeeding at 12 months of age. Ethiopia has attained the 2030 global exclusive breastfeeding target and the WHO recommendation for continued breastfeeding. There needed to be better quality of complementary food at 12 months of age. Infants had a low dietary diversity with a poor intake of micronutrient‐rich food, and many had sugary food or beverages. This food pattern could increase the risk of undernutrition, including micronutrient deficiencies. Sugary food consumption may contribute to later overweight and obesity. Enhancing access to facility‐ and community‐based infant breastfeeding and complementary feeding education interventions is essential to strengthen and maintain early initiation of breastfeeding, exclusive breastfeeding at 5 months of age, continued breastfeeding at 12 months, and substantially increase the quality of complementary food. Efforts to counteract the aggressive marketing of unhealthy food could also reduce infants' consumption of sugary foods or beverages. Intensified efforts to reduce national poverty and food insecurity are critical to improving infants' dietary diversity and nutrient‐dense food consumption through increasing affordability or household access to safe and nutritious foods.

## Author Contributions

A.T., L.Å.P., J.S., S.L., D.B., A.D., T.G., Z.A., T.G., G.T., T.M., and S.S. contributed to the conceptualization of the study. A.T. analysed and interpreted the data and drafted the manuscript. A.T., L.Å.P., J.S., S.L., D.B., A.D., T.G., Z.A., T.G., G.T., S.S., and M.Z. contributed to the analysis and writing of the paper. All authors read and approved the final manuscript.

## Conflicts of Interest

The authors declare no conflicts of interest.

## Supporting information


**Supplementary Table 1.** Characteristics of participating and non‐participating infants, PMA Ethiopia panel study, July 2020 to August 2021.

**Supplementary Table 2.** Sensitivity analysis of feeding practices for participating and non‐participating infants, PMA Ethiopia panel study, July 2020 to August 2021.

## Data Availability

All data used in this publication are publicly available at https://www.pmadata.org/data/request-access-datasets.
