# Supplementary material for: Infant Feeding Practices in Ethiopia: Birth Cohort Study in Five Regions
Source: Matern Child Nutr. 2025 Jan 31;21(2):e13804. doi: 10.1111/mcn.13804 (PMC11956073; doi:10.1111/mcn.13804)
Supplement: Supplementary file 1 — Supplementary Table 1. Characteristics of participating and non‐participating infants, PMA Ethiopia panel study, July 2020 to August 2021. [file MCN-21-e13804-s001.docx]

Supplementary Table 1. Characteristics of participating and non-participating infants, PMA Ethiopia panel study, July 2020 to August 2021

|  | Participating infants (n=1,850) | Non-participating infants (n=454) | Chi-square |
| --- | --- | --- | --- |
| Characteristics | Unweighted n (%) | Unweighted n (%) | P-value |
| Region |  |  |  |
| Afar | 178 (10) | 62 (14) | <0.001 |
| Amhara | 413 (22) | 50 (12) |  |
| Oromia | 539 (29) | 140 (32) |  |
| SNNP^*^ | 506 (27) | 123 (28) |  |
| Addis Ababa | 214 (12) | 60 (14) |  |
| Residence |  | N=435 |  |
| Urban | 640 (35) | 190 (44) | <0.001 |
| Rural | 1,210 (65) | 245 (56) |  |
| Household wealth | N=1,849 |  |  |
| 1 (Poorest) | 353 (19) | 69 (15) | 0.022 |
| 2 | 297 (16) | 71 (16) |  |
| 3 | 312 (17) | 59 (13) |  |
| 4 | 331 (18) | 96 (21) |  |
| 5 (Wealthiest) | 556 (30) | 159 (35) |  |
| Education | N=1,849 | N=454 |  |
| No schooling | 753 (41) | 157 (35) | 0.038 |
| Primary school | 671 (36) | 169 (37) |  |
| Secondary | 241 (13) | 80 (18) |  |
| Technical and vocational | 78 (4) | 24 (5) |  |
| Higher education | 106 (6) | 24 (5) |  |
| Number of antenatal care visits (n=1,848) | N= 1,848 | N=454 |  |
| 0 | 962 (52) | 257 (57) | 0.001 |
| 1 | 274 (15) | 38 (8) |  |
| 2 | 234 (13) | 48 (11) |  |
| 3 | 174 (9) | 58 (13) |  |
| ≥4 | 204 (11) | 53 (12) |  |
| Received postnatal care within two days |  |  |  |
| No postnatal care | 1,497 (81) | 400 (88) | 0.002 |
| Received ≤2 days | 79 (4) | 17 (4) |  |
| 3-7 days | 156 (9) | 22 (5) |  |
| 8-28 days | 118 (6) | 15 (3) |  |

*Southern Nations Nationalities and Peoples
