# Supplementary material for: Infant Feeding Practices in Ethiopia: Birth Cohort Study in Five Regions
Source: Matern Child Nutr. 2025 Jan 31;21(2):e13804. doi: 10.1111/mcn.13804 (PMC11956073; doi:10.1111/mcn.13804)
Supplement: Supplementary file 2 — Supplementary Table 2. Sensitivity analysis of feeding practices for participating and non‐participating infants, PMA Ethiopia panel study, July 2020 to August 2021. [file MCN-21-e13804-s002.docx]

Supplementary Table 2. Sensitivity analysis of feeding practices for participating and non-participating infants, PMA Ethiopia panel study, July 2020 to August 2021

| Infant feeding practices | Participating infants (n=1,850) | Participating and non-participating infants (n=2,304) | P-value^E^ |
| --- | --- | --- | --- |
|  | Weighted % (95% CI) | Weighted % (95% CI) |  |
| Early initiation of breastfeeding | 67 (62, 71) | 66 (62, 70) | 0.73 |
| Exclusive breastfeeding at five months of age | 69 (67, 71) | 69 (66, 71) | 0.54 |
| Continued breastfeeding at 12 | 97 (96, 98) | 97 (97, 98) | 0.15 |
| Complementary feeding at 12 months of age | | |  |
| Animal protein intake^J^ | 55 (51, 60) | 48 (44, 52) | <0.001 |
| Minimum diet diversity^H^ | 16 (13, 19) | 16 (13, 19) | 0.93 |
| Vitamin A rich food intake | 62 (58, 66) | 55 (50, 59) | <0.001 |
| Iron rich diet food | 6 (4, 8) | 5 (4, 7) | <0.001 |
| Zero fruit or vegetable | 65 (61, 69) | 70 (66, 73) | <0.001 |
| Sugary food or beverage | 49 (44, 55) | 43 (38, 48) | <0.001 |

^E^ Chi-square test; ^J^Flesh, dairy and egg; ^H^Five or more food groups
